# Spatial and Bidirectional Work Function Modulation of Monolayer Graphene with Patterned Polymer “Fluorozwitterists”

**DOI:** 10.1021/acscentsci.4c00704

**Published:** 2024-08-06

**Authors:** James Nicolas Pagaduan, Nicholas Hight-Huf, Le Zhou, Nicholas Dix, Uvinduni I. Premadasa, Benjamin Doughty, Thomas P. Russell, Ashwin Ramasubramaniam, Michael Barnes, Reika Katsumata, Todd Emrick

**Affiliations:** † Polymer Science and Engineering Department, University of Massachusetts, Amherst, Massachusetts 01003, United States; ‡ Department of Chemistry, University of Massachusetts, Amherst, Massachusetts 01003, United States; ⊥ Department of Mechanical and Industrial Engineering and Materials Science Graduate Program, University of Massachusetts, Amherst, Massachusetts 01003, United States; ∥ Chemical Sciences Division, Oak Ridge National Laboratory, Oak Ridge, Tennessee 37831, United States; § Materials Sciences Division, Lawrence Berkeley National Laboratory, Berkeley, California 94720, United States

## Abstract

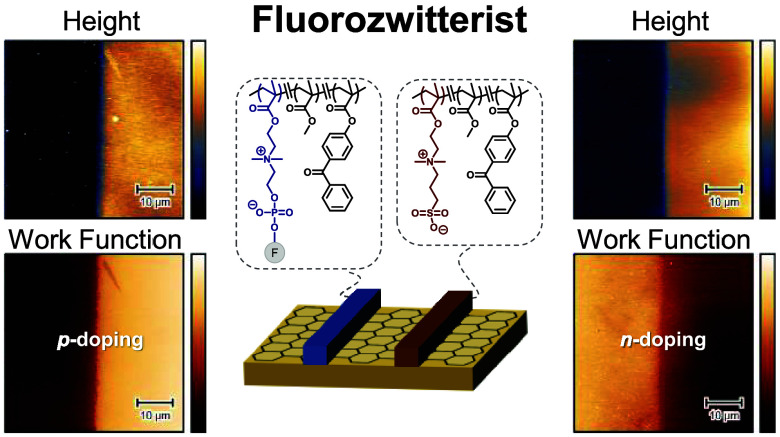

Understanding the electronic properties resulting from soft–hard material interfacial contact has elevated the utility of functional polymers in advanced materials and nanoscale structures, such as in work function engineering of two-dimensional (2D) materials to produce new types of high-performance devices. In this paper, we describe the electronic impact of functional polymers, containing both zwitterionic and fluorocarbon components in their side chains, on the work function of monolayer graphene through the preparation of negative-tone photoresists, which we term “fluorozwitterists.” The zwitterionic and fluorinated groups each represent dipole-containing moieties capable of producing distinct surface energies as thin films. Kelvin probe force microscopy revealed these polymers to have a *p*-doping effect on graphene, which contrasts the work function decrease typically associated with polymer-to-graphene contact. Copolymerization of fluorinated zwitterionic monomers with methyl methacrylate and a benzophenone-substituted methacrylate produced copolymers that were amenable to photolithographic fabrication of fluorozwitterist structures. Consequently, spatial alteration of zwitterion coverage across graphene yielded stripes that resemble a lateral *p*-*i*-*n* diode configuration, with local increase or decrease of work function. Overall, this polymeric fluorozwitterist design is suitable for enabling simple, solution-based surface patterning and is anticipated to be useful for spatial work function modulation of 2D materials integrated into electronic devices.

## Abstract 2

The fluorozwitterist macromolecular design enables the use of a single-component polymer overlayer for simple photopatterning of the work function of graphene to resemble a *p*-*i*-*n* diode configuration.

## Introduction

Work function (WF) engineering of two-dimensional (2D) materials constitutes an effective route to enhanced performance of electronic devices.^1^ The WF of a conducting material, defined as the energy required to promote an electron from Fermi level to vacuum, can be tailored to facilitate charge injection, alter band alignment, and, for 2D materials, control the nature and extent of doping.^2−4^ For monolayer graphene, strategies to modulate WF include chemical modification, application of mechanical strain, and electrostatic gating.^5−9^ Another prominent approach involves synthetic polymers, which are attractive for their solution processability, chemical functionality, film-forming capabilities, and utility in lithographic patterning.^10−12^ In particular, polymer zwitterions, which possess pendent groups with covalently connected cationic and anionic components, are recognized as useful for their orthogonal solubility (advantageous for layer-by-layer deposition), electrical neutrality (lacking mobile counterions that may interrupt stability and performance), and antifouling behavior (reducing protein adsorption that can degrade bioelectronic devices).^13−15^ Our prior studies uncovered the substantial WF shifts of metal electrodes and 2D materials induced by polymer zwitterions, which led to enhanced performance in field-effect transistors and solar cells,^16−18^ where electronic response arises from the dipole moments associated with the physisorbed zwitterions.^19^

In earlier studies on photopatternable polymer zwitterions on graphene, the introduction of sterically bulky groups, such as piperidine, on the ammonium cation of the sulfobetaine (SB) zwitterion led to enhanced WF reduction (i.e., *n*-type doping).^20^ Density functional theory (DFT) calculations pointed to the importance of the normal vs. transverse components of the zwitterion dipole relative to the underlying graphene layer (noting that the WF shift is directly proportional to the normal component of the dipole) and that the steric footprint of piperidine may orient the SB cation away from the underlying graphene and toward surface normal orientation. That is, polymers with pendent zwitterions oriented normal to graphene produce larger WF shifts than those with transverse orientations, which agreed qualitatively with experimental results obtained by ultraviolet photoelectron spectroscopy (UPS). Further, owing to the presence of benzophenone-containing comonomers, these zwitterionic polymers proved useful as negative-tone photoresists, or “zwitterists”, amenable to photo-cross-linking in areas of UV-exposure and fabrication of graphene-based field-effect transistor (FET) devices. We subsequently probed the electronic influence of other polymer zwitterions in contact with graphene, such as poly(2-methacryloyloxyethyl phosphorylcholine) (PMPC).^21^ Scanning probe evaluation of both the polymer and graphene sides of the polymer-on-graphene construct revealed that polarization, rather than pure charge transfer, is principally responsible for graphene doping. Although the zwitterionic dipoles of PMPC are oriented in a direction opposite to that of the SB-methacrylate polymer backbone, both structures led to *n*-doping, noting that calculations inferred that steric factors of the cationic moieties of the pendent PC groups push them away from the surface. Overall, these prior studies set forth exciting opportunities for WF modulation through variation of zwitterion chemical structure that may alter molecular orientation.

The chemical versatility of polymers encourages tethering of other dipole-rich and surface-active functionalities, beyond hydrocarbons, to modulate electronic properties. For example, we successfully embedded fluorocarbons directly into choline phosphate (CP) monomers, which upon polymerization afforded fluorinated choline phosphate (FCP)-based polymer zwitterions.^22^ Surface grafting of FCP polymers revealed contact angles and surface energies intermediate between those of PMPC and conventional fluorinated polymers,^23^ with large contact angle hysteresis values pointing to dynamic reorganization in response to the contacting fluid. Given the electron-withdrawing character and control of molecular orientation imparted by the fluorocarbons, we sought to investigate the potential for electronic interactions resulting from physisorption of FCP polymers on graphene. The strong orientational driving forces associated with the surface energy of fluorinated groups^24,25^ and their reported role as *p*-dopants of 2D materials^26,27^ further motivate the studies described below.

We specifically describe the electronic impact of polymeric FCPs on the WF of monolayer graphene to generate negative-tone “fluorozwitterists.” Notably, these unusual polymer zwitterions have two types of dipoles embedded in their side chains, each with distinct contributions to surface energy: one arising from the zwitterionic CP groups and the other from the fluorinated alkyl groups. Kelvin probe force microscopy (KPFM) of FCP-coated graphene revealed electronic characteristics indicative of *p*-doping of graphene. Sum-frequency generation (SFG) vibrational spectroscopy provided chemical and structural insights into how the dipoles orient at the polymer–graphene interface. Moreover, the preparation and use of FCP-containing copolymers with methyl methacrylate (MMA) and a benzophenone-substituted methacrylate (BPMA) gave a route to robust film formation and photo-cross-linking, both valuable features for lithographic patterning. By patterning FCP polymers with PSBMA on the same graphene substrate via sequential UV-lithography, neighboring zwitterionic stripes were prepared that resemble a *p*-type/intrinsic/*n*-type (*p*-*i*-*n*) diode configuration laterally across graphene. Overall, the knowledge gained from this study holds promise for fine-tuning the electronic characteristics of 2D materials-based devices, including the spatial patterning of *p*- or *n*-type character by simple contact with dipole-rich moieties.

## Results and Discussion

### Polymer Synthesis and Electronic Characterization

**Figure 1:**
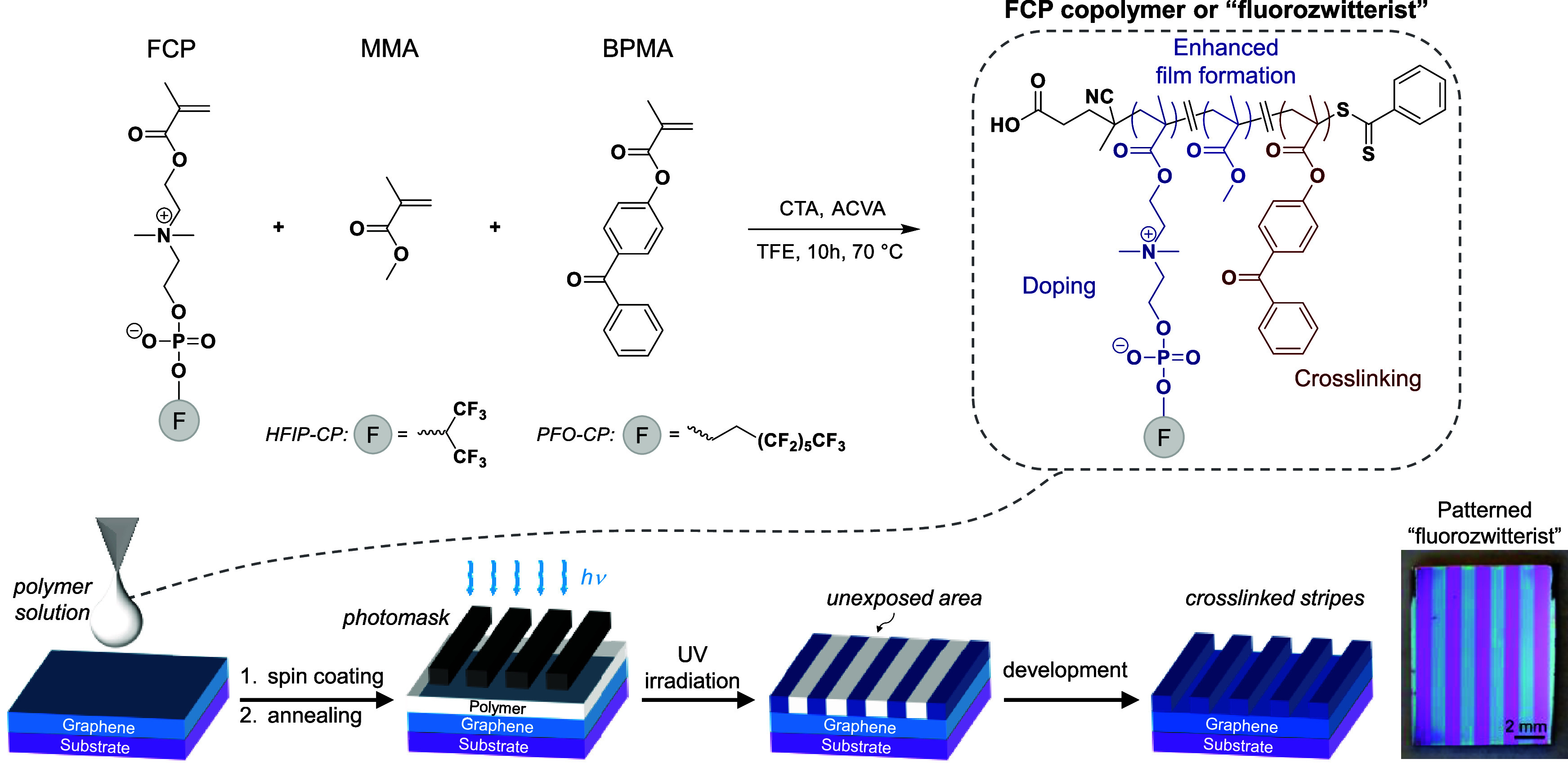
Top: Synthesis of fluorinated zwitterion-based random copolymers by RAFT polymerization with variation of fluorinated (F) groups; Bottom: lithographic patterning of “fluorozwitterist” on graphene through a chrome-coated photomask for spatial electronic tuning; Far-right: photograph of actual 1000-μm wide patterned stripes (blue) on graphene/SiO_2_/Si substrate (purple).

**Figure 2:**
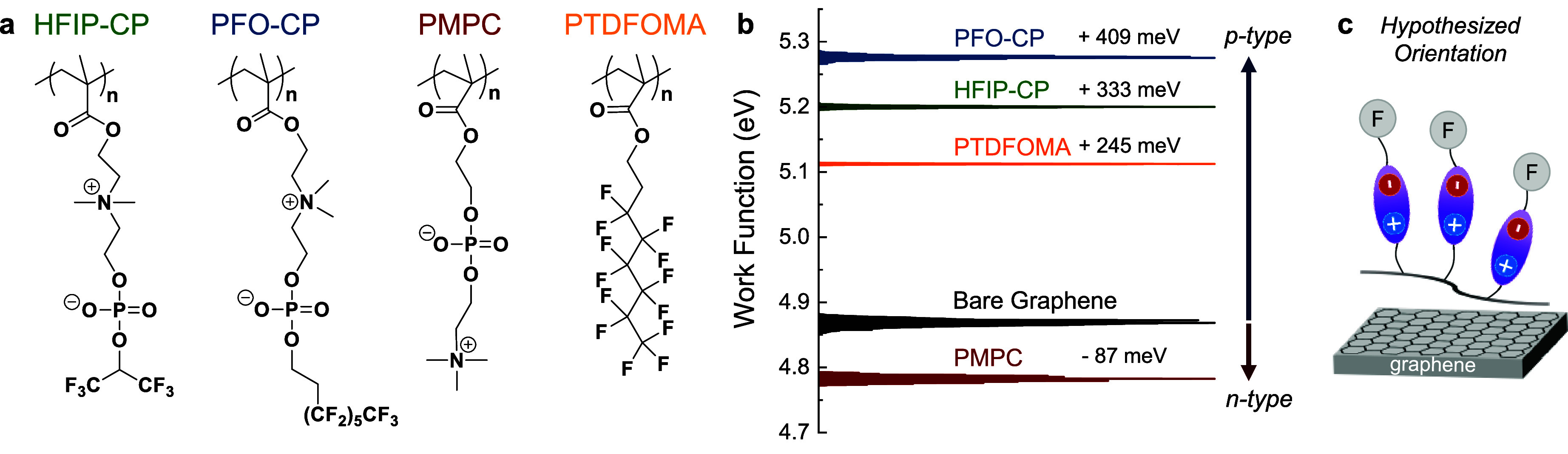
(a) Chemical structures of homopolymers of 1,1,1,3,3,3-hexafluoro-2-propanol-substituted choline phosphate (HFIP-CP) and 1*H*,1*H*,2*H*,2*H*-perfluoro-1-octanol-substituted choline phosphate (PFO-CP), poly(2-methacryloyloxyethyl phosphorylcholine) (PMPC), and poly(tridecafluoro-*n*-octyl methacrylate) (PTDFOMA); (b) Work function distributions derived from KPFM of homopolymers on graphene/SiO_2_/Si deposited from 1 mg/mL solutions; and (c) Illustration of hypothesized orientation of pendent FCP group with respect to graphene.

The functional polymers utilized in lithographic patterning and fundamental investigations were synthesized by controlled free radical polymerization of FCP monomers with MMA and BPMA. This yielded polymers amenable to photolithography in a similar fashion as the “zwitterist” macromolecular design^20^ by employing a photo-cross-linkable monomer with this new class of fluorinated zwitterions.^22^ Specifically, the FCP monomers in Figure 1 were employed in copolymerizations using reversible addition–fragmentation chain transfer (RAFT) polymerization at 70 °C in a 2,2,2-trifluoroethanol (TFE) solution containing a dithiobenzoate chain transfer agent and an azo-initiator. This afforded the desired random copolymers with estimated number-average molecular weight (*M*_n_) in the 11–20 kDa range and polydispersity index (*Đ*) of 1.1–1.2. Their corresponding ^1^H NMR spectra (Figure S1 and S3) showed distinct signals from each repeat unit, enabling calculation of polymer composition (50:44:6 and 53:41:6 FCP:MMA:BPMA for a typical example of HFIP-CP and PFO-CP copolymers, respectively) that was in good agreement with the feed ratio employed (50:45:5). In addition, homopolymers of HFIP-CP and PFO-CP were prepared to probe the impacts of the FCP moieties without the influence of MMA and BPMA. To gain insight into the distinct contributions of each dipole type, homopolymers of PMPC and the nonzwitterionic fluorinated methacrylate termed PTDFOMA were also prepared, with the collection of structures shown in Figure 2a (and characterization data given in Figures S5–S8 and Table S1).

To assess the electronic influence of polymer films on graphene, dual-pass KPFM was employed as a scanning probe technique to measure the electric force between a conductive microcantilever and a grounded sample.^28^ KPFM characterizes the impact of the environment on the electronic properties of 2D materials, such as from physisorbed surface dopants.^10,11,20,21,29−31^ In dual-pass KPFM, surface topography was determined during the first pass, followed by a second pass to measure surface potential contrast (SPC). Changes in WF are derived from SPC by calibrating the WF of the probe with a highly oriented pyrolytic graphite (HOPG) reference. In our experiments, polymer films on graphene/SiO_2_/Si substrates were applied by spin-coating from 1 mg/mL TFE solutions at 4000 rpm. Spectroscopic ellipsometry revealed polymer layers with an estimated thickness of ∼3–4 nm (Figure S9). KPFM indicated a marked difference in doping effects imparted by the four homopolymers, as depicted in Figure 2b, with the corresponding height and WF images given in Figure S10. Notably, PMPC was the only polymer to induce work function *reduction*, by ∼87 meV, which agrees qualitatively with our prior work.^21^ In contrast, the fluorine-containing polymers resulted in a WF increase of graphene, indicative of a *p*-doping effect elicited by the polymer coating. Specifically, PTDFOMA, HFIP-CP, and PFO-CP homopolymers led to WF increases of ∼245, ∼333, and ∼409 meV, respectively. While *p*-type doping induced by PTDFOMA may be anticipated from the presence of the electron-withdrawing fluorocarbon structure, the FCP homopolymers produced the largest WF shifts despite having fewer (HFIP) or identical (PFO) numbers of fluorine atoms as PTDFOMA. This apparent synergistic electronic action between the zwitterionic and fluorinated groups in contact with graphene inverts the sign of Δϕ relative to PMPC and augments the magnitude relative to PTDFOMA. This finding may be rationalized by considering the potential orientation of the FCPs, where localization of the fluorocarbon components to the air interface drives zwitterion orientation such that the cationic moiety points toward graphene (Figure 2c). In contrast, in our prior work, calculations indicated that the zwitterionic moieties in PMPC and PSBMA prefer to physisorb in a nearly flat orientation on graphene, which maximizes attractive dispersion interactions.^20,21^ Thus, we suggest that the competition between the energetics of zwitterion physisorption on graphene and segregation of the fluorocarbon component to the air interface may ultimately control FCP orientation.

While KPFM measures a relative energy difference in work function between a conductive tip and an underlying substrate, UPS directly probes the electronic levels in the valence band by measuring the kinetic energy of emitted photoelectrons following UV absorption under ultrahigh vacuum.^32^ Specifically, the energy difference between the incident photons (21.2 eV for He I radiation) and that corresponding to the secondary electron cutoff in the obtained spectra represents the WF shift. To prevent charging (i.e., blocking collection of low kinetic energy electrons upon photoelectron emission), a bias voltage is normally applied, which necessitates use of conductive samples. Here, a graphene/Au/Ti/SiO_2_/Si configuration was employed as the substrate, where the Au underlayer enhances sample conductivity and minimizes charging. Furthermore, since the typical information depth for UPS is 2–3 nm, very thin polymer films were prepared from 0.1–0.5 mg/mL polymer solutions on Au-coated wafers (measured as <2 nm by spectroscopic ellipsometry) (Figure S9). These thickness values agree with the step-heights obtained from atomic force microscopy (AFM) measurements performed on scratched polymer-coated graphene/SiO_2_/Si samples (Figure S11).

**Figure 3:**
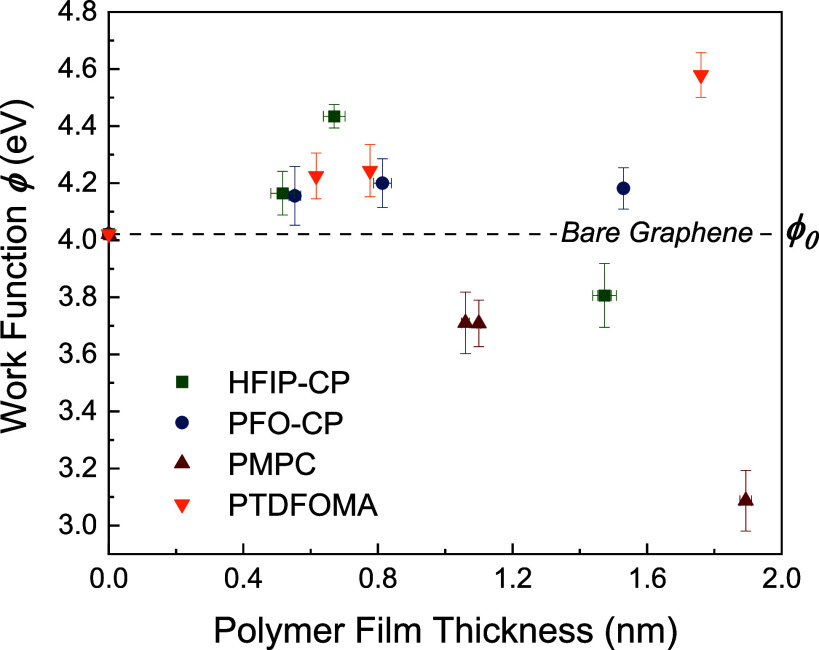
Comparison of WF values of polymer-coated graphene measured by UPS (mean ± SD of *n* = 4 to 10 replicates).

As shown in Figure 3, polymer film thickness influenced the magnitude of WF shifts^20^ relative to bare graphene measured at 4.02 eV (denoted by horizontal line). When coated with PMPC and PTDFOMA, the measured WF shifted to lower and higher values, respectively, in agreement with KPFM measurements. The magnitudes increased with film thickness, with respective shifts of −0.93 and 0.56 eV observed as PMPC and PTDFOMA films approached 2 nm. As such, polymer film thickness provides a tunable knob for electronic modulation as may be desirable for a given application. In particular, the electron-withdrawing groups of PTDFOMA induced an increase in hole density, shifting the Fermi level of graphene closer to the valence band, and increasing the energy required to promote an electron to vacuum.^33^ On the other hand, calculations suggest that the anionic component of MPC (with a μ_⊥_ equal to 0.52 D) points toward graphene,^21^ which should decrease the vacuum level and consequently reduce WF, in accord with studies on polymer zwitterion-coated metals^34^ and organic–metal interfaces.^35^

Both HFIP-CP and PFO-CP coatings produced WF increases, especially for thinner films, with magnitudes comparable to PTDFOMA, confirming the *p*-doping behavior of FCP polymers. Interestingly, for the thickest films shown in Figure 3, WF shifts were smaller, which we attribute to charging (accumulation of surface charge upon electron emission). Very thick films (>10 nm) led to unreasonable WF values upon bias correction (i.e., the correction factor should be less than the applied voltage to set the Fermi level to zero). While at this stage we do not understand the work function finding for HFIP-CP at ∼1.5 nm film thickness (deviating from the other data points), we speculate that the lower degree of fluorination of this polymer may reduce the influence of orientation as film thickness increases. Notably, UPS relies on the kinetic energy of emitted photoelectrons to detect electron energy loss due to work required when passing through the polymer layer. Moreover, although both UPS and KPFM provide information on electronic levels, UPS measures area-average WF while KPFM scans the local WF with high spatial resolution. Thus, UPS is sensitive to surface contamination (imperfections or pinholes), and as such complementary KPFM measurements of thicker films are useful to understand if surface imperfections have impacted UPS measurements. Intrinsic defects that may arise within the lattice structure of graphene can impact the electronic properties and should be considered as well.^36,37^ Additional contributions from stacked dipoles, involving multiple layers of zwitterions, may also play a role, though at present we have no evidence to support the formation of such structures. Irrespective of the exact mechanisms responsible for the effects, the electronic characterization demonstrated to this point shows that altering zwitterion chemistry enables control over doping behavior on graphene, from *n*-type to *p*-type, and thus in principle sets up a fabrication strategy for in-plane device construction.

### Probing Interfacial Configuration by Sum-Frequency Generation Vibrational Spectroscopy

To probe the distribution and packing of chemical moieties adsorbed on graphene, we employed sum-frequency generation (SFG) vibrational spectroscopy. Based on symmetry arguments, SFG signals are *interface-specific*, allowing one to gain chemical and structural insights into buried interfaces that are otherwise inaccessible to conventional linear spectroscopies. Particularly, we sought to understand the effect of the pendent group functionality on the organization of the polymer backbone when interacting with graphene, and to relate these structural motifs to the change in work function. Details of the measurements are given in the Methods section.

**Figure 4:**
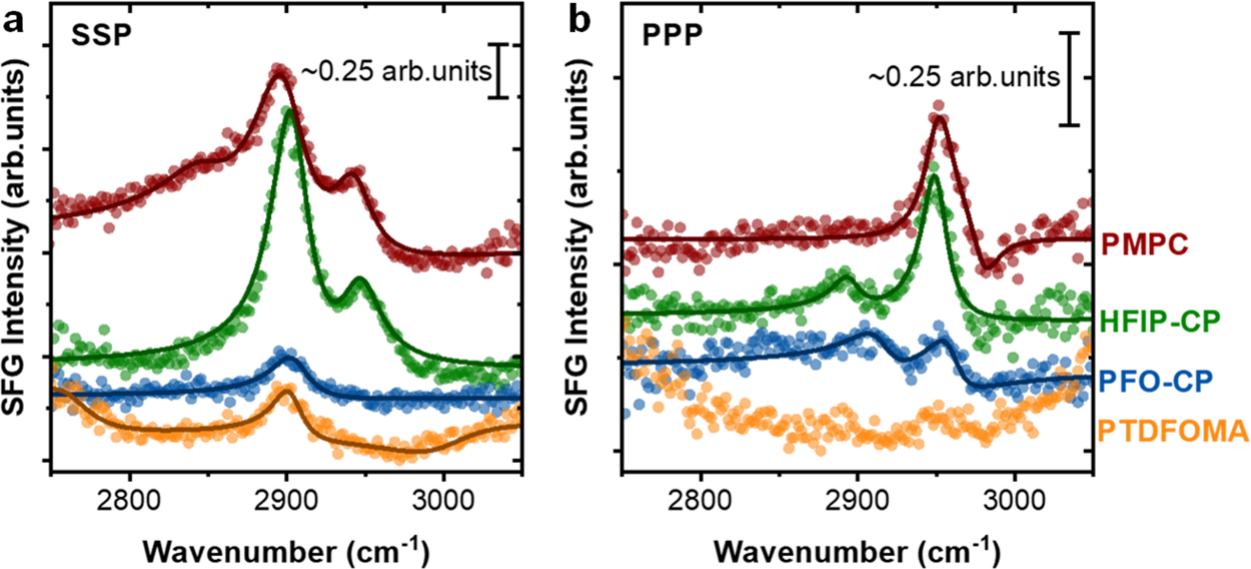
SFG spectra obtained from PMPC, HFIP-CP, PFO-CP, and PTDFOMA polymer thin films deposited on graphene/SiO_2_ (quartz) at (a) SSP and (b) PPP polarization combinations. The spectra are offset for clarity.

Figure 4 displays the SFG spectra obtained from PMPC, HFIP-CP, PFO-CP, and PTDFOMA homopolymers adsorbed on graphene/SiO_2_ (quartz) at SSP and PPP polarization combinations. The solid curves correspond to fits to the experimental data points obtained using eq 1 in the Methods section. The data in this frequency region report on CH vibrational modes from the methacrylate backbone and the various pendent arms, as assigned below. Control experiments were performed with thicker polymer films (∼1 μm) deposited on SiO_2_ to identify signals arising from the air–polymer interface (Figure S12a). The control spectra clearly differ from those obtained from thin (4–5 nm) polymer–graphene (Figure 4) and polymer–SiO_2_ interfaces (Figure S12b), indicating that the SFG signals for thin polymer coatings correspond to the polymer–substrate interface and that the supporting substrate strongly affects polymer organization at the buried interface.

In all SSP spectra presented in Figure 4a, the prominent feature at 2900 cm^–1^ is assigned to the symmetric stretch (-*ss*) from the methyl group (−CH_3_) on the methacrylate chain.^38−40^ The broad peak at 2950 cm^–1^ in the SSP spectra of both HFIP-CP and PMPC is attributed to a Fermi resonance with unresolved contributions from out-of-phase −CH_3_ asymmetric stretches (-*as*).^38,40^ These asymmetric stretching modes are evident in the PPP spectra (Figure 4b), in agreement with SFG selection rules. Additionally, the PMPC SSP spectrum shows broad unresolved features at lower wavenumbers (2845 to 2865 cm^–1^) that arise from methylene symmetric stretches in the methacrylate backbone (C–CH_2_) and the pendent arms (O–CH_2_/N–CH_2_).^38,39^ These CH_2_ bands are not detected in the SSP spectra from other polymers, indicating that (1) the presence of fluorinated groups in the pendent arms influences the overall methacrylate backbone conformation and (2) the pendant arms themselves orient in unique ways depending on the chemical makeup. Supporting this, we identified C–CH_2_-*as* (2895 cm^–1^) and weaker O–CH_2_-*as*/N–CH_2_-*as* (2915 cm^–1^) modes in the PPP spectra of HFIP-CP and PFO-CP polymers, respectively.^38^

To further corroborate the qualitative structural differences observed with different pendant groups, control experiments were performed in which the polymers were deposited on SiO_2_ coverslips without graphene (Figure S12b). Here, the polymer chains contact a polar surface environment (in contrast to the nonpolar graphene or air), which should result in distinct interfacial conformations. Indeed, the spectral profiles at SiO_2_ interfaces show clear differences compared to those at graphene interfaces in Figures 4 and S12a, indicating unique interactions of the pendant arms with the polar SiO_2_ surface and different associated backbone conformations. Interestingly, all spectra from SiO_2_ interfaces have observable contributions from CH_2_ groups (both backbone and pendent groups), whereas the prominent −CH_3_-*ss* resonance found in graphene samples appears as a weak shoulder. This means that the polymer backbone and pendent groups arrange differently at graphene vs. polar interfaces, thereby permitting modulation of the work function by differential packing and associated dipolar couplings.^41^ For instance, at graphene interfaces, PTDFOMA polymers with longer fluorinated chains result in a poorly ordered interface, as evidenced by weak overall SFG signals (Figure 4).^42−44^ Similarly, the PFO-CP samples exhibit poor overall signal and ordering, indicating that longer pendant arms with increasing fluorine content generally disrupt interfacial polymer packing. We note that the interfacial ordering at graphene interfaces, as qualitatively gauged by changes in SFG response, does not map one-to-one onto the measured work function shifts. That is, while interfacial order goes as HFIP-CP > PMPC > PFO-CP ∼ PTDFOMA, only PMPC was found to reduce the work function of graphene.

**Figure 5:**
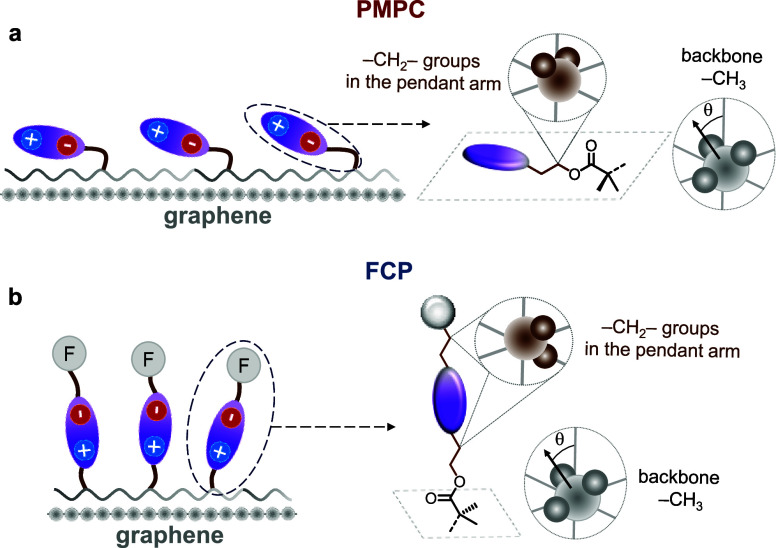
Schematic diagram of the hypothesized orientations of (a) PMPC and (b) FCP polymer chains adsorbed on graphene based on SFG findings. For clarity, methylene groups in the pendant arm are drawn in brown. Pendant arm orientations with respect to graphene corroborate the measured work function shifts.

To more quantitatively elucidate the impact of orientation on work function, we performed numerical analysis, as previously reported,^38,45,46^ with the parameters used detailed in the Supporting Information. We did not obtain any orientational information for PTDFOMA, due to the weak signals in the PPP polarization combination. Taking the CH_3_-*as*,_PPP_/CH_3_-ss,_SSP_ amplitude ratios for the other three polymers, the average tilt angle of the −CH_3_ group of the methacrylate backbone with respect to the surface normal is in the range of 31–37° for PMPC and FCP, as represented by θ in Figure 5. These results suggest that the fundamental interactions between the graphene monolayer and the nonpolar *backbone* remain unchanged when varying pendant arms, but that the interfaces become increasingly disordered.

Similarly, we can interpret the arrangement of the CH_2_ groups in the pendant arm by noting their intensities; here, the CH_2_ groups on the PMPC pendent arms are generally tilted away from the surface plane, thus resulting in stronger peaks in the SSP spectra. This results in a whole pendant arm orientation that is parallel to the surface plane, as illustrated in Figure 5a. In contrast, for HFIP-CP and PFO-CP polymers, the lack of this signal from CH_2_ groups suggests that these groups are oriented parallel to the surface plane, inducing the whole pendant arm to stand more upright in a bottlebrush-like configuration, as shown in Figure 5b. The different orientations of the pendant arms should result in different dipolar couplings between the zwitterion and graphene. Those polymers with fluorinated tails correspond to systems with larger work functions, where the zwitterion orientation is likely to be aligned out of the interfacial plane. For PMPC, the only sample that produced a decrease in work function, the pendant arms orient such that the zwitterion would be more closely parallel to the interface, in agreement with calculations.^21^ Based on the SFG results and relating them to the hypothesized orientation of pendent arms illustrated in Figure 2c, we find that the presence of a longer fluorinated side chain results in poorer backbone packing, presumably mediated by pendant arms. These pendant groups containing the zwitterionic moiety then have different couplings with graphene based on their absolute orientation with respect to the surface.

### Lithographic Patterning of Fluorozwitterists

**Figure 6:**
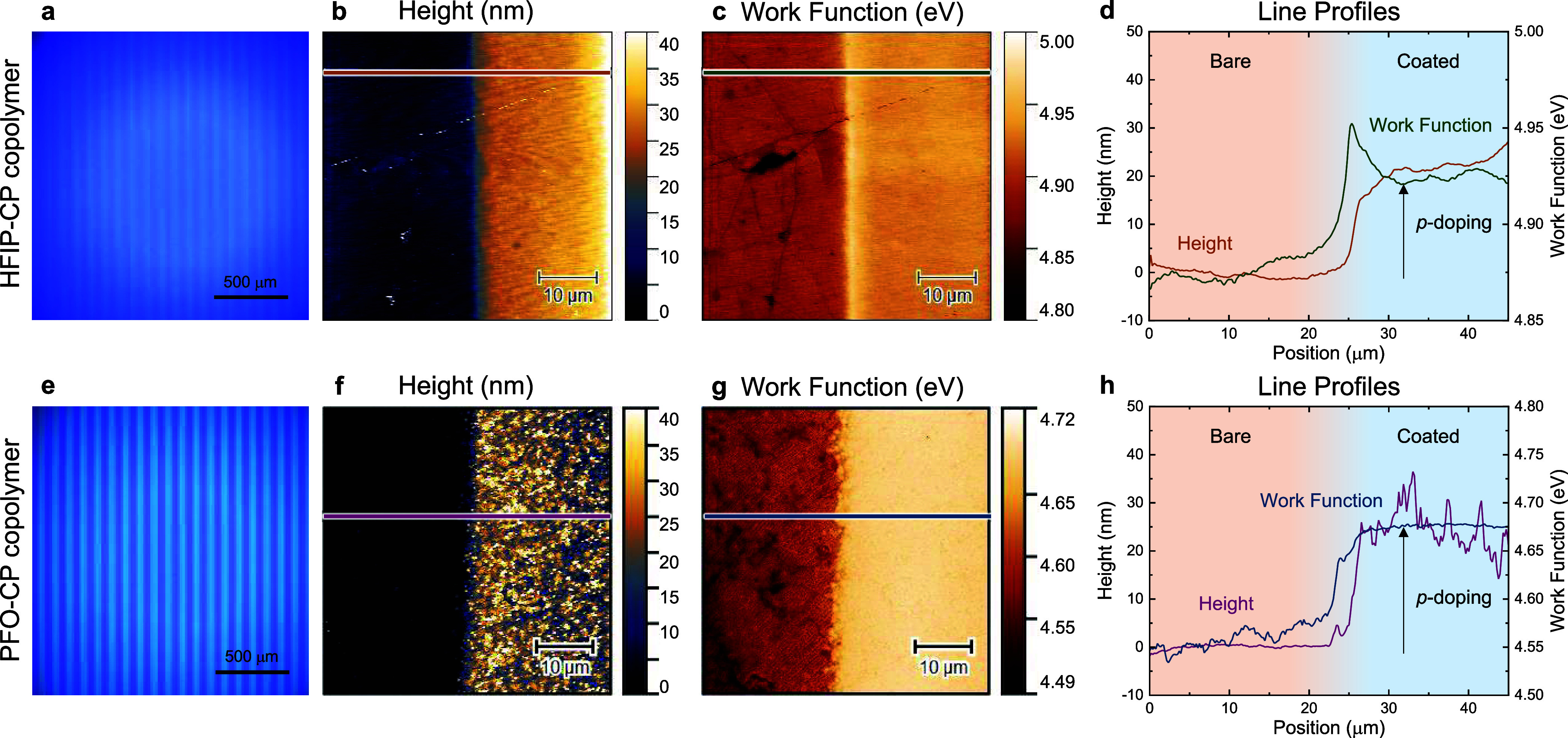
(a,e) Optical microscopy of HFIP-CP and PFO-CP copolymers with 50 μm patterned stripes on graphene/SiO_2_/Si substrate. Kelvin probe force microscopy of the interface between patterned stripes and bare graphene showing the (b,f) height and (c,g) work function images with corresponding (d,h) line profiles. Both polymers induced *p*-doping relative to bare graphene surface.

The prepared copolymers of fluorinated zwitterions with MMA and BPMA were then utilized as negative-tone photoresists or “fluorozwitterists.” Polymer solutions in TFE were similarly spin-coated on graphene/SiO_2_/Si substrates, yielding films of 20–30 nm thickness. Figure 1 illustrates the photolithographic process for patterning fluorozwitterists on graphene, accomplished by UV irradiation at 365 nm with an energy dose of 20,000 mJ cm^–2^ through a chrome-coated quartz photomask, followed by developing in TFE to remove un-cross-linked regions, and drying under N_2(g)_. Lithographic patterning was successful on films prepared from 5 mg/mL polymer solutions; employing more dilute solutions led to either no stripe formation or the appearance of thin, nonuniform features (Figure S14). Both random copolymers produced uniform stripes of 20–30 nm thickness, with pitch of ∼50 μm, as shown in Figure 6, indicating good pattern fidelity relative to the mask employed. Dual-pass KPFM enabled topographic and electronic evaluation of patterned polymer–graphene interfaces (Figure 6b,c,f,g). Although both stripes comprised only ∼50 mol % of the fluorinated zwitterions, the *p*-doping behavior was evident. A sharp WF increase over a distance of ∼1–2 μm was quantified across the graphene interfaces with both fluorozwitterists (Figure 6d,h). Specifically, the patterned HFIP-CP and PFO-CP, with RMS surface roughness of ∼2 and ∼15 nm, induced WF shifts of ∼50 and ∼120 meV, respectively, in qualitative agreement with the WF shifts for the corresponding homopolymers. Despite the roughness of the PFO-CP stripe, the WF changes remained smooth. Notably, the unpatterned PFO-CP homopolymer did not exhibit such surface roughness (Figure S10) and any roughness observed is not due to polymer crystallinity, as differential scanning calorimetry confirmed their amorphous nature (Figure S15). Thus, surface roughness likely resulted from the lithographic process.

### Co-patterning of Fluorozwitterist and Zwitterist

**Figure 7:**
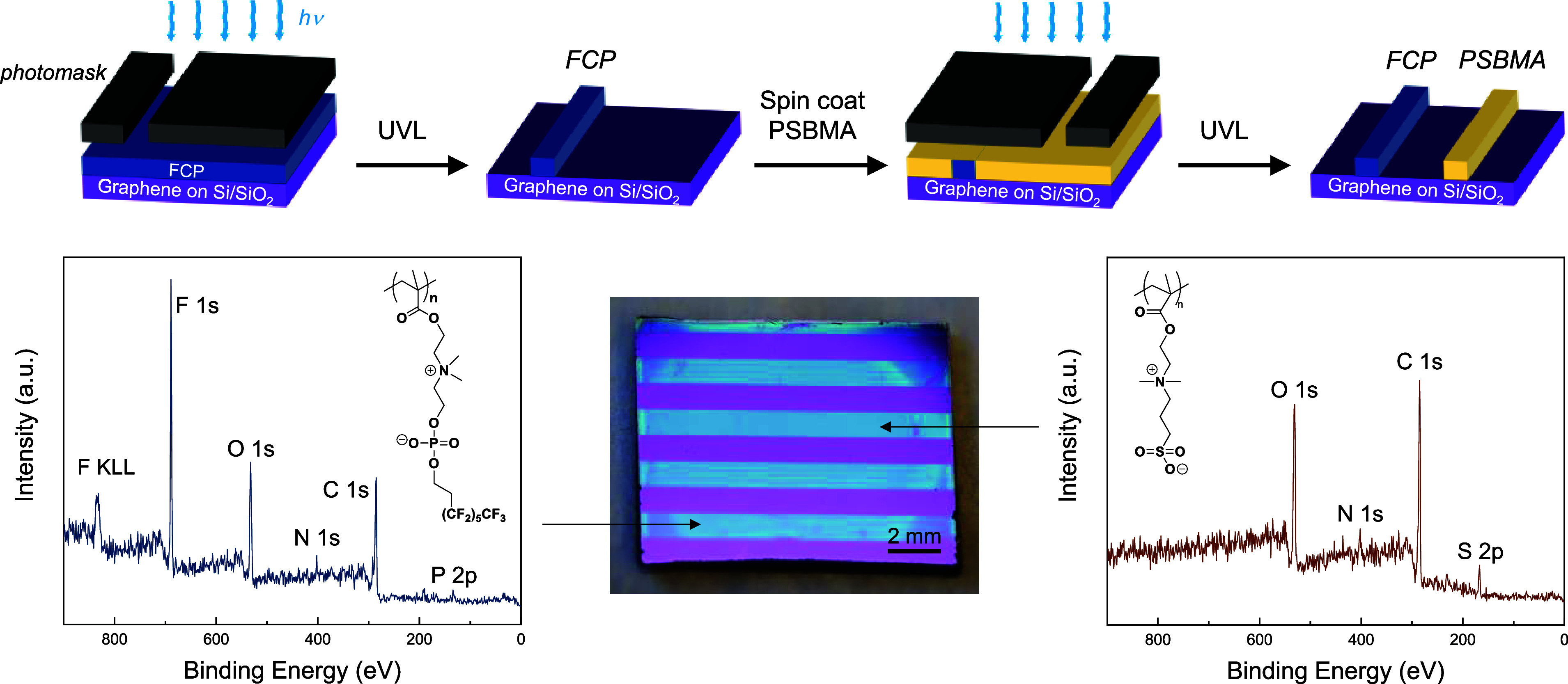
Top: Schematic diagram for copatterning polymeric fluorozwitterist (FCP) and zwitterist (PSBMA) on the same graphene substrate via sequential UV lithography; Bottom: XPS survey spectra of individual patterned polymer stripes (blue) on graphene (purple) indicated on the photograph with relevant peaks corresponding to the dipole-doping moieties.

Considering our findings to this point, we hypothesized that graphene doping may be spatially modulated by lateral patterning of different polymer zwitterions. To test this, a random copolymer of SBMA with MMA and BPMA (Figure S16) was employed as the solution-processable zwitterist. Figure 7 exhibits the simple process for copatterning distinct polymer zwitterions via masked UV lithography. First, a solution of the FCP-based random copolymer was spin-coated on a graphene/SiO_2_/Si substrate. The sample was dried under vacuum at 40 °C prior to photo-cross-linking at 365 nm with a dose of 20,000 mJ cm^–2^ through a chrome-coated quartz photomask. The mask was positioned so as to block a region of the first coating from being cross-linked and provide space for the next polymer zwitterion to be patterned. After UV exposure, the wafer was soaked in TFE for 10 s to remove the un-cross-linked regions and dried with a stream of N_2(g)_. Then, a second solution containing PSBMA copolymer was spin-coated on the partially patterned graphene/SiO_2_/Si substrate. Repeating the exposure and development procedures yielded neighboring stripes of fluorozwitterist and zwitterist. X-ray photoelectron microscopy (XPS) analysis revealed excellent fidelity of the patterned regions, in which the expected elements were observed in the stripes of FCP (without sulfur contamination) and PSBMA (without fluorine or phosphorus contamination).

**Figure 8:**
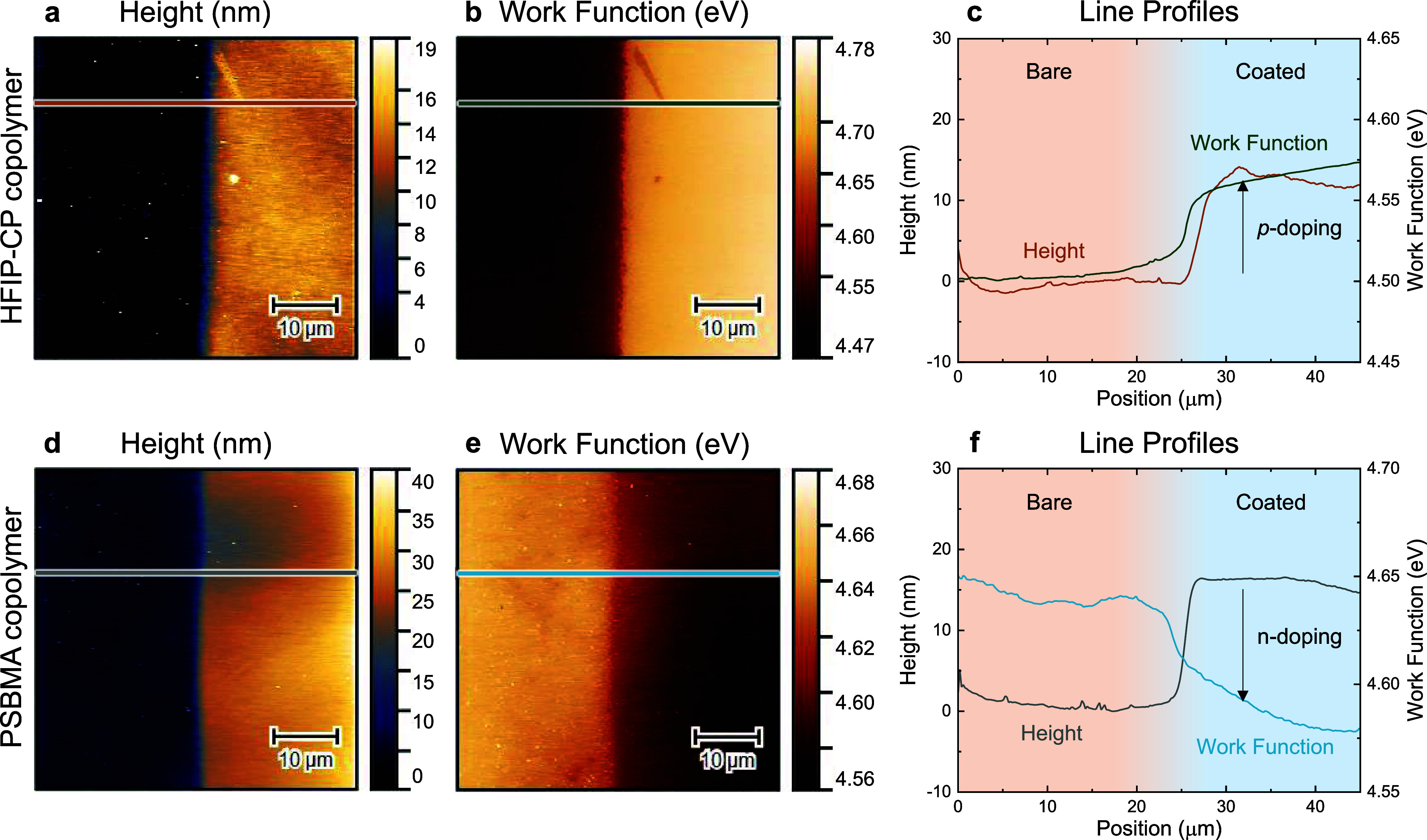
Co-patterned HFIP-CP (fluorozwitterist) and PSBMA (zwitterist) copolymers: KPFM of the interface between patterned stripes and bare graphene showing the (a,d) height and (b,e) work function images with corresponding (c,f) line profiles. The patterned fluorozwitterist and zwitterist induced *p*- and *n*-doping, respectively, relative to neighboring bare graphene surface.

KPFM measurements validated our hypothesis and uncovered *local WF shifts on the same substrate relative to nearby bare graphene regions* induced by the FCP (WF increase) and PSBMA (WF decrease). As seen in Figure 8, the HFIP-CP and PSBMA copolymer patterns correspondingly shifted the WF of graphene by 75 meV (*p*-doping) and −70 meV (*n*-doping), wherein the neighboring stripes resemble a lateral *p*-*i*-*n* diode configuration. This structure has increasingly gained attention for low-power, high-speed optoelectronics, wherein the intrinsic region induces a lateral built-in electric field that is essential for ultrafast and efficient separation of photogenerated carriers.^47,48^ The *p*-*i*-*n* configuration is useful for expanding the utility of graphene, which inherently has a low on/off current ratio due to the absence of a bandgap. Common approaches to spatially control WF of graphene include substitutional doping,^49^ patterned coating,^50^ and layering.^51^ A strategy more conceptually related to our method involve patterning of self-assembled monolayers (SAMs), wherein CH_3_-terminated SAMs neutralized the *p*-doping behavior of the SiO_2_ substrate while NH_2_-terminated SAMs *n*-doped graphene.^6^ However, this requires silane coupling agents and a photoresist to grow and pattern SAMs on Si, followed by pattern transfer, whereas the fluorozwitterist design simplifies the fabrication process since the polymer acts simultaneously as the coating, resist, and dopant, thus bypassing the etching and transfer steps.

We also observed the importance of the patterning order: thinner films of the fluorozwitterists with larger electronic shifts were realized when FCP was patterned first, followed by PSBMA. In contrast, the WF shifts were less significant when FCP patterning followed PSBMA (Figures S17–19 and Table S2). Since XPS, which probes the stripes to ∼10 nm depth, provided evidence for the generation of discrete polymer stripes, the impact of patterning order may suggest some degree of FCP-PSBMA mixing at the graphene–polymer interface, though we have not probed this specifically. Nonetheless, the patternability of these polymers provides an opportunity to produce devices with two laterally adjacent or remote polymers in contact with the 2D material to elicit local surface potential modification and form homojunctions while preserving the structural and electronic properties of graphene. Investigating the electronic performance of copatterned graphene devices is central to our future work. The simple, solution-based lithographic strategy we introduced herein holds great potential for spatially controlling the nature and extent of doping on 2D surfaces with arbitrarily predetermined patterns and promising lateral resolution.

## Conclusions

We demonstrated *p*-doping of graphene by its interfacial contact with fluorinated polymer zwitterions and provided fundamental insights into the role of zwitterionic dipole orientation. Interestingly, the WF shift induced by the fluorinated polymer zwitterions surpassed the shifts caused by a more conventional methacrylic fluoropolymer. By simple solution-based copatterning of different polymer zwitterions, both positive and negative WF shifts were measured on the same graphene substrate, conceptually resembling a lateral *p*-*i*-*n* diode configuration. The knowledge gained from this study provides insights for the field of 2D materials-based devices by elucidating the importance of tailored hard–soft 2D-polymer interfaces, in which polymer chemistry is combined with patterning methodology to afford access to spatially controlled doping of 2D electronic structures.

## Methods

### Polymer Synthesis and Characterization

Copolymers of FCP-substituted methacrylate, MMA and BPMA were prepared by reversible addition–fragmentation chain-transfer (RAFT) polymerization as shown in Figure 1. The chain transfer agent 4-cyano-4-(phenylcarbonothioylthio)pentanoic acid, 4,4-azobis(4-cyanovaleric acid) (ACVA) initiator ([CTA]:[ACVA] = 1:0.2), FCP monomer (50 equiv), MMA (45 equiv), BPMA (5 equiv) and TFE (1.6 M with respect to total monomer amount) were mixed in a 7 mL vial wrapped with aluminum foil. The reaction mixture was degassed using dry N_2(g)_ for 30 min and then stirred at 70 °C for 10 h. The polymerization was quenched by removal from heat source and exposure to air. Triple precipitation in diethyl ether, water, and diethyl ether followed by drying in vacuo afforded the solid polymer product (∼50% yield). The homopolymers were synthesized following similar procedures without the addition of MMA and BPMA. ^1^H, ^19^F, and ^31^P NMR (500 MHz) spectra of the polymer products were recorded on a Bruker Ascend 500 spectrometer equipped with a Prodigy cryoprobe. Gel permeation chromatography (GPC, using PMMA calibration standards) was conducted using an eluent mixture of TFE with 0.02 M sodium trifluoroacetate at 40 °C on an Agilent 1200 system equipped with the following: an isocratic pump operated at 1 mL/min, a degasser, an autosampler, one 50 mm × 8 mm PSS PFG guard column (Polymer Standards Service), and three 300 mm × 7.5 mm PSS PFG analytical linear M columns with 7 μm particle size (Polymer Standards Service), and an Agilent 1200 refractive index detector.

### Polymer Film Sample Preparation

CVD monolayer graphene was prepared and transferred onto a 4-in. Au/Ti/SiO_2_/Si wafer by Grolltex, Inc. (San Diego, CA). The substrate was then cut into 1 cm × 1 cm pieces. Solutions of FCP homopolymers as well as PMPC and PTDFOMA with varying concentrations, from 0.1 to 1.0 mg/mL, were individually spin-coated onto these substrates at 500 rpm for 5 s and then at 4000 rpm for 55 s. Prior to deposition, the polymer solutions were vortexed and filtered through a polytetrafluoroethylene (PTFE) membrane (0.2 μm VWR). To remove residual solvent, the film samples were dried under vacuum at 40 °C overnight prior to electronic and spectroscopic characterizations.

### Lithographic Patterning

Solutions of FCP-based random copolymers in TFE with an optimal concentration of 5 mg/mL were individually spin-coated onto graphene/SiO_2_/Si substrates at 500 rpm for 5 s and then at 4000 rpm for 55 s. Prior to deposition, the polymer solutions were vortexed and filtered through a PTFE membrane (0.2 μm VWR). To remove residual solvent, samples were dried under vacuum at 40 °C overnight prior to lithography. The polymer films were cross-linked by UV irradiation (λ = 365 nm) with a dose of 20,000 mJ cm^–2^ through a chrome-coated quartz photomask. The samples were soaked in TFE for 10 s to remove the un-cross-linked regions and dried with a nitrogen gun, affording patterned resists. For copatterned resists, the same procedure was performed twice with 4 mg/mL of PSBMA copolymer in TFE as the additional polymer solution. Optical imaging of patterned samples was conducted using a ZEISS Axioscope 5 microscope with Axiocam 305 color camera.

### Polymer Film Thickness Measurement

The same homopolymer solutions as described above, with varying concentrations, were separately spin-coated on Au substrates at 500 rpm for 5 s and then 4000 rpm for 55 s. Polymer film thickness values were estimated by ellipsometry using a J.A. Woollam RC2 spectroscopic ellipsometer at varying angles of incidence (45°, 50°, 55°, 60°, 65°). The values were calculated by fitting the experimental data with the Cauchy equation: *n* = *A* + *B*/λ^2^ where *n* is the refractive index, λ is the light wavelength in μm, and *A* and *B* are constants with value of 1.5 and 0.01, respectively.

### Kelvin Probe Force Microscopy (KPFM)

KPFM data were collected on a Digital Instruments Bioscope AFM/KPFM in two-pass lift mode under ambient atmospheric conditions (22 °C, 45% RH). The AFM probes were platinum/iridium-coated silicon (SCM-PIT-V2) with f_0_ of ∼70 kHz, used as supplied by Bruker. Samples were grounded using copper tape from Electron Microscopy Sciences. Work function was calculated by measuring a freshly cleaved highly oriented pyrolytic graphite (HOPG, ZYB grade, Bruker) reference sample (with a work function of 4.65 eV) to calibrate the tip’s work function, which was then used to relate the measured surface potential to the corresponding work function. The profiles were analyzed using the scanning probe microscopy data analysis software Gwyddion.

### Ultraviolet Photoelectron Spectroscopy (UPS) and X-ray Photoelectron Spectroscopy (XPS)

Both UPS and XPS were carried out using a Thermo Scientific Nexsa Surface Analysis System with a He I discharge line (21.2 eV) and monochromatic aluminum Kα X-ray source (1486.6 eV), respectively. For UPS, a −10 V sample bias was applied to collect the low kinetic energy electrons. All data were obtained with a pass energy of 2.0 V and a step size of 0.05 eV at a base pressure of 5 × 10^–7^ millibar or lower. The UPS spectra reported were averaged from three scans. For XPS, the flood gun was turned on during all measurements to prevent charging. All data were collected using a 72-W focused X-ray beam with a spot size of 50 μm at a base pressure of 5 × 10^–7^ millibar or lower. Survey scans were obtained with a pass energy of 200 eV and a step size of 1 eV. The work function values from UPS and elemental compositions from XPS were calculated using the Thermo Avantage software package (v5.9925).

### Sum-Frequency Generation (SFG) Vibrational Spectroscopy

The SFG samples were prepared using the homopolymer solutions and spin-coating procedure described above. Here, ∼ 4 nm thick polymer films were deposited on graphene/SiO_2_ (quartz) and SiO_2_ (control) surfaces. SFG experiments were performed using a home-built SFG spectrometer reported previously.^52−54^ SFG signals were collected in a reflection geometry, where colinearly propagating narrowband near-infrared (NIR, λ_NIR_ ∼ 803 nm) and broadband mid-infrared (IR, λ_IR_ ∼ 3390 nm) beams were temporally and spatially overlapped at the polymer samples at a 60° angle relative to the surface normal. Samples were placed such that the polymer films were facing upright, and the graphene layer buried beneath. Control experiments on graphene-free samples used the same geometry but variable polymer layer thicknesses. Polarizations for both NIR and IR beams were varied with half-waveplates and the radiated SFG signal was polarization resolved using an achromatic half-waveplate/polarizer pair. Letters describing SFG polarization combinations (e.g., SSP) denote the polarization state of the SFG, NIR, and IR fields, respectively. Single frame exposure times of 3 min were used for measurements and were averaged over 3 frames. Background-subtracted SFG spectra were scaled by the response from gold films collected in the PPP combination. Intensity spectra were fit using
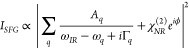
1where *I*_*SFG*_ is the measured SFG intensity,  is the nonresonant background and ϕ is the phase angle. Amplitudes (*A*_*q*_), resonant frequencies (*ω*_*q*_), and peak widths (*Γ*_*q*_) are the parameters for the fit vibrational modes.^52−55^ A summary of all fitting parameters is provided in the Supporting Information.

## Supporting Information Available

Synthetic methods and characterization of the monomers and polymers; ellipsometry data; supporting SFG spectra, fitting results, details on orientational analysis; supplementary KPFM and AFM profiles, DSC curves, and optical images (PDF)
