# Supplementary material for: Spatial and Bidirectional Work Function Modulation of Monolayer Graphene with Patterned Polymer “Fluorozwitterists”
Source: ACS Cent Sci. 2024 Aug 6;10(8):1629–39. doi: 10.1021/acscentsci.4c00704 (PMC11363338; doi:10.1021/acscentsci.4c00704)
Supplement: Supplementary file 1 — oc4c00704_si_001.pdf [file oc4c00704_si_001.pdf]

SUPPORTING INFORMATION

Spatial and Bidirectional Work Function Modulation of Monolayer Graphene with Patterned Polymer "Fluorozwitterists"

James Nicolas Pagaduan,[†] Nicholas Hight-Huf,[‡] Le Zhou,[†] Nicholas Dix,[‡] Uvinduni I. Premadasa,[||] Benjamin Doughty,[||] Thomas P. Russell,[†§] Ashwin Ramasubramaniam,[⊥] Michael Barnes,[‡] Reika Katsumata,*[†] and Todd Emrick*[†]

[†]*Polymer Science and Engineering Department,* [‡]*Department of Chemistry, and* [⊥]*Department of Mechanical and Industrial Engineering and Materials Science Graduate Program, University of Massachusetts, Amherst, MA 01003, USA*

[||]*Chemical Sciences Division, Oak Ridge National Laboratory, Oak Ridge, TN 37831, USA*

[§]*Materials Sciences Division, Lawrence Berkeley National Laboratory, Berkeley, CA 94720, USA*

**E-mail of corresponding authors: tsemrick@mail.pse.umass.edu; rkatsumata@umass.edu*

A. Materials

Acetonitrile (anhydrous, 99.8%), 4,4′-azobis(4-cyanovaleric acid) (ACVA, 98%), 4-cyano-4-(phenylcarbonothioylthio)pentanoic acid (CTA, >97%), 2-(dimethylamino)ethyl methacrylate (DMAEMA, 98%), 2-methacryloyloxyethyl phosphorylcholine (MPC, 97%), [2-(methacryloyloxy)ethyl]dimethyl-(3-sulfopropyl)ammonium hydroxide (SBMA, 95%), methyl methacrylate (MMA, 99%), 4-hydroxybenzophenone (98%), methacryloyl chloride (97%), triethylamine (TEA, 99.0+%), gold-coated silicon wafers (99.999% Au, layer thickness 1000 Å, 99.99% Ti adhesion layer), and 3 Å molecular sieves were purchased from Sigma Aldrich (St. Louis, MO, USA). Before use, MPC was washed with diethyl ether and dried *in vacuo*, TEA was distilled over calcium hydride and TDFOMA and MMA were purified by passage through a plug of basic alumina. 1*H*,1*H*,2*H*,2*H*-Tridecafluoro-*n*-octyl methacrylate (TDFOMA, >98.0%, stabilized with HQ and MEHQ) was obtained from TCI Chemical (Portland, OR, USA). Diethyl ether (anhydrous, ≥99.0%) and 4-methoxyphenol (99%) were purchased from Fisher Scientific (Waltham, MA, USA). Ethylene chlorophosphate (ECP, 95%) was purchased from Alfa Aesar (Haverhill, MA, USA) and purified by Kugelrohr distillation. 1,1,1,3,3,3-Hexafluoro-2-propanol (HFIP, 99.5%), 2,2,2-trifluoroethanol (TFE, 99.9%), and 1*H*,1*H*,2*H*,2*H*-perfluoro-1-octanol (PFO) were obtained from Oakwood Products, Inc (West Columbia, SC, USA). Chloroform-*d* (99.8%), methanol-d_4 (99.8%) and 2,2,2-trifluoroethanol-d_3 (99%) were purchased from Cambridge Isotope Laboratories (Tewksbury, MA, USA). CVD-grown monolayer graphene on quartz (1 cm × 1 cm) and monolayer graphene on Si/SiO_2 wafer (4 inches) were obtained from ACS Material, LLC (Pasadena, CA, USA) and Grolltex, Inc (San Diego, CA, USA), respectively. Chrome-coated quartz photomask with a critical dimension of 5.0 μm and tolerance of 2.0 μm was purchased from PhotoSciences, Inc. (Torrance, CA, USA). The fluorinated zwitterionic monomers (HFIP-CP and PFO-CP) and benzophenone methacrylate (BPMA) were synthesized according to reported procedures.[1,2]

B. Supplementary Data

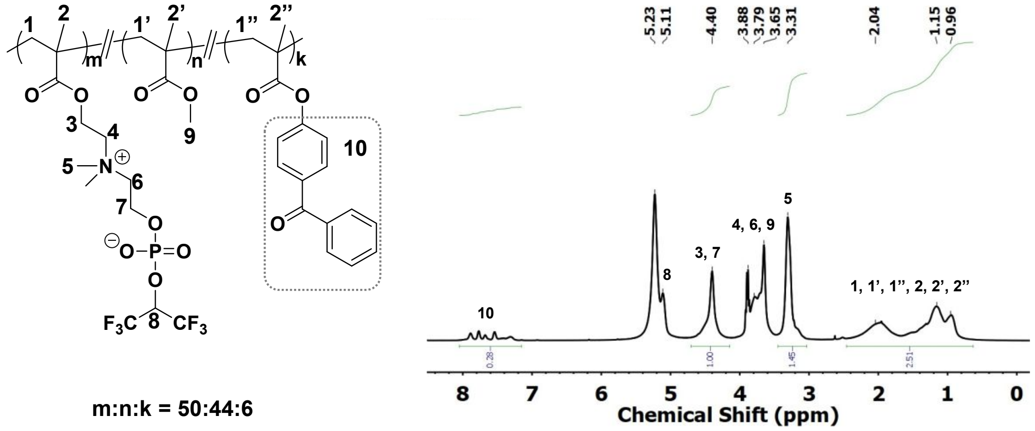

Figure S1. Chemical structure and ^{1}H NMR spectrum of HFIP-CP copolymer (TFE-d_3, 500 MHz).

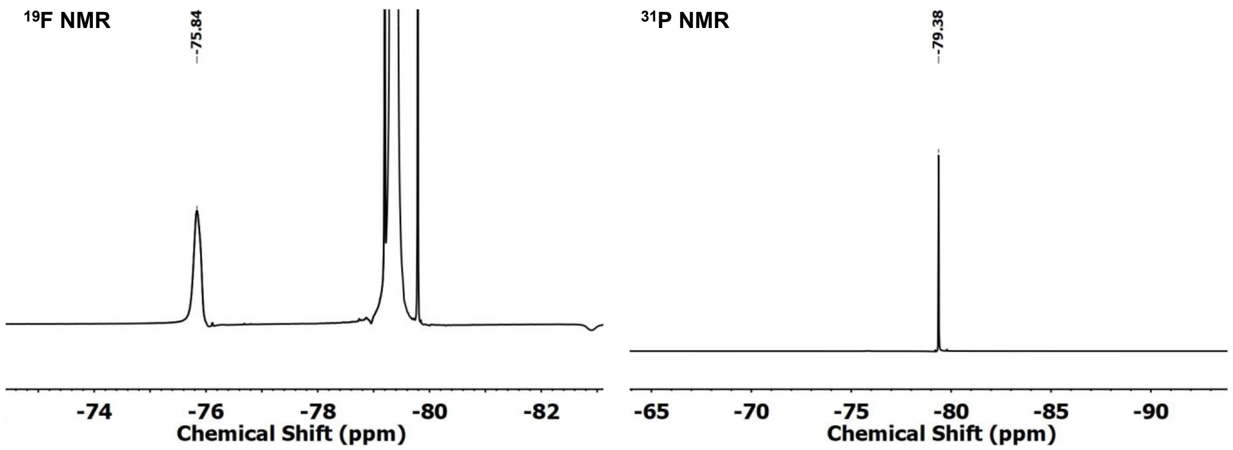

Figure S2. ^{31}P and ^{19}F NMR spectra of HFIP-CP copolymer (TFE-d_3, 500 MHz).

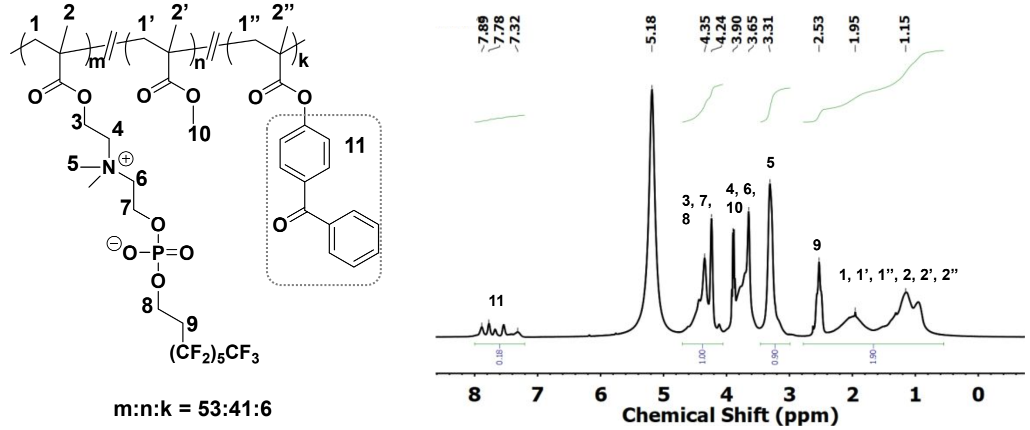

Figure S3. Chemical structure and ^{1}H NMR spectrum of PFO-CP copolymer (TFE-d_3, 500 MHz).

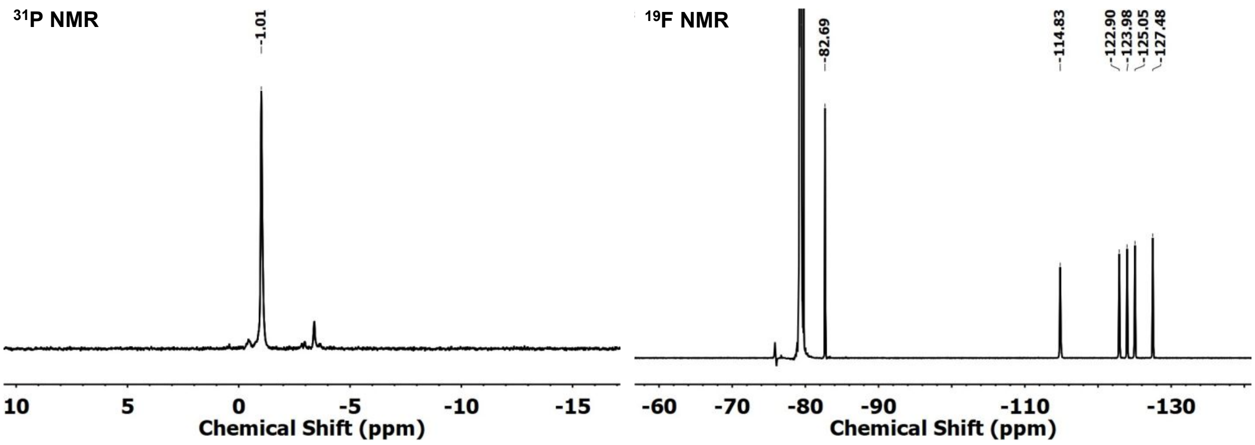

Figure S4. ^{31}P and ^{19}F NMR spectra of PFO-CP copolymer (TFE-d_3, 500 MHz).

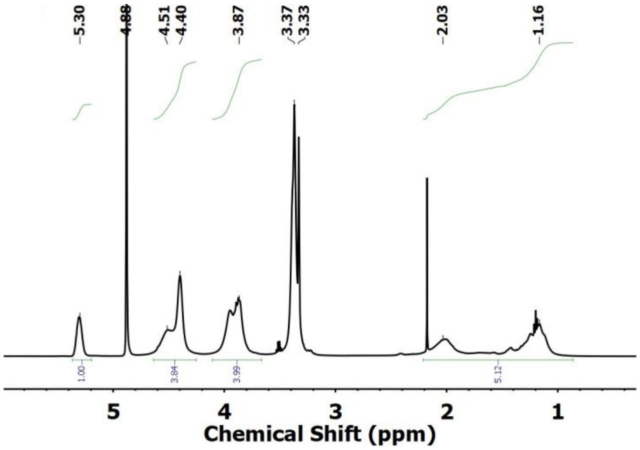

Figure S5. ^{1}H NMR spectrum of HFIP-CP homopolymer (CD_3OD, 500 MHz).

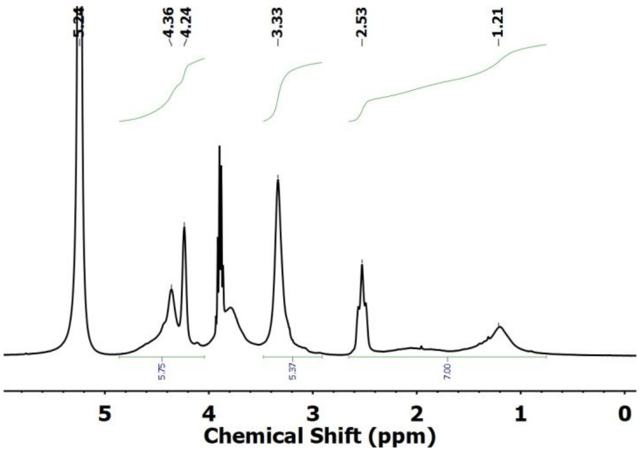

Figure S6. ^{1}H NMR spectrum of PFO-CP homopolymer (TFE-d_3, 500 MHz).

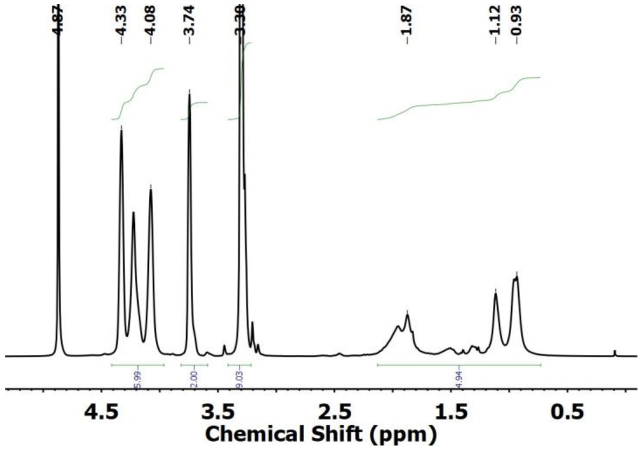

Figure S7. ^{1}H NMR spectrum of PMPC homopolymer (CD_3OD, 500 MHz).

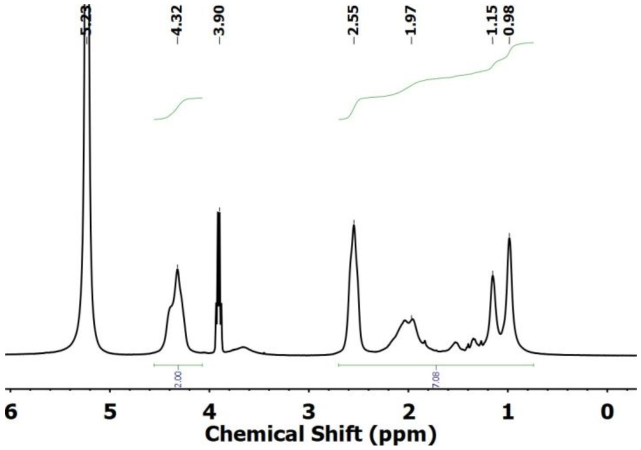

Figure S8. ^{1}H NMR spectrum of PTDFOMA homopolymer (TFE-d_3, 500 MHz).

Table S1. Characterization data for FCP-based polymers and control samples.

Polymer	Feed Ratio [Zwitterion]:[MMA]:[BPMA]	Actual Ratio[a]	M_n[b] (kDa)	M_w[b] (kDa)	Đ[b]
HFIP-CP copolymer	50:45:5	50:44:6	11.6	12.3	1.06
PFO-CP copolymer	50:45:5	53:41:6	20.0	23.8	1.19
HFIP-CP homopolymer	---	---	6.8	8.7	1.28
PFO-CP homopolymer	---	---	12.1	13.9	1.15
PMPC homopolymer	---	---	27.8	29.7	1.07
PTDFOMA homopolymer	---	---	6.6	7.2	1.09
PSBMA copolymer	50:45:5	45:51:4	19.1	21.0	1.10

a) Determined by ^1H NMR spectroscopy; b) Estimated by GPC relative to PMMA standards, eluting in TFE

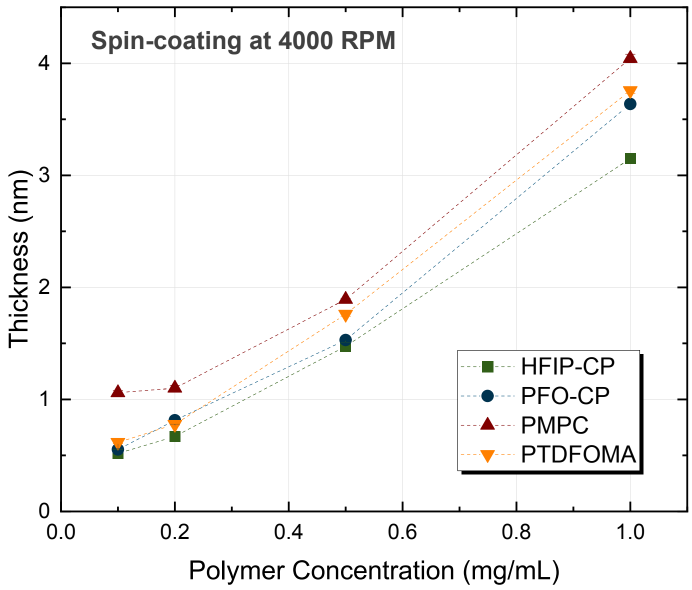

Figure S9. Results of ellipsometry-derived thickness measurements of polymer coatings on Au.

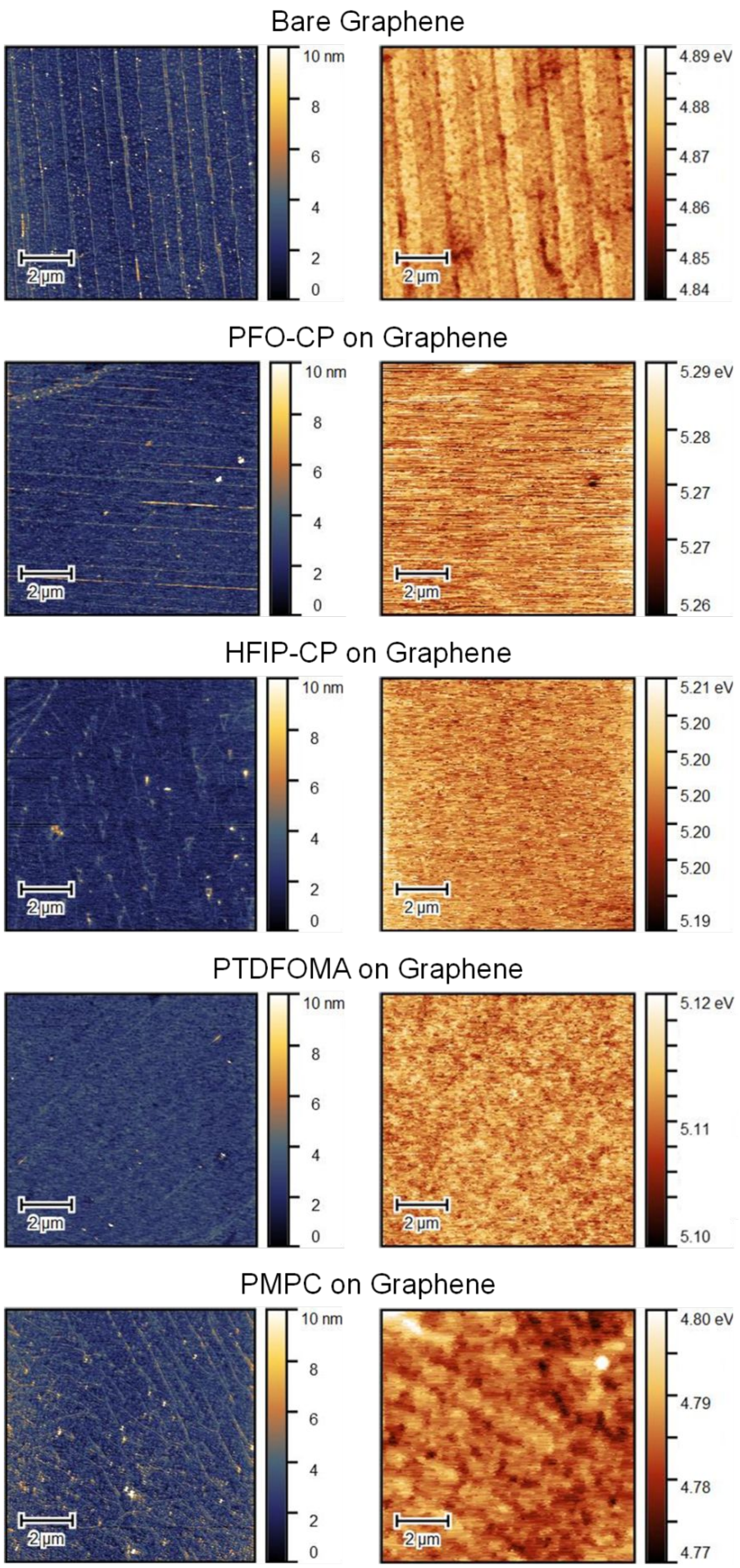

Figure S10. Kelvin probe force microscopy of bare and homopolymer-coated graphene showing the height (left) and work function (right) images. All fluorinated polymers induced p-doping of graphene.

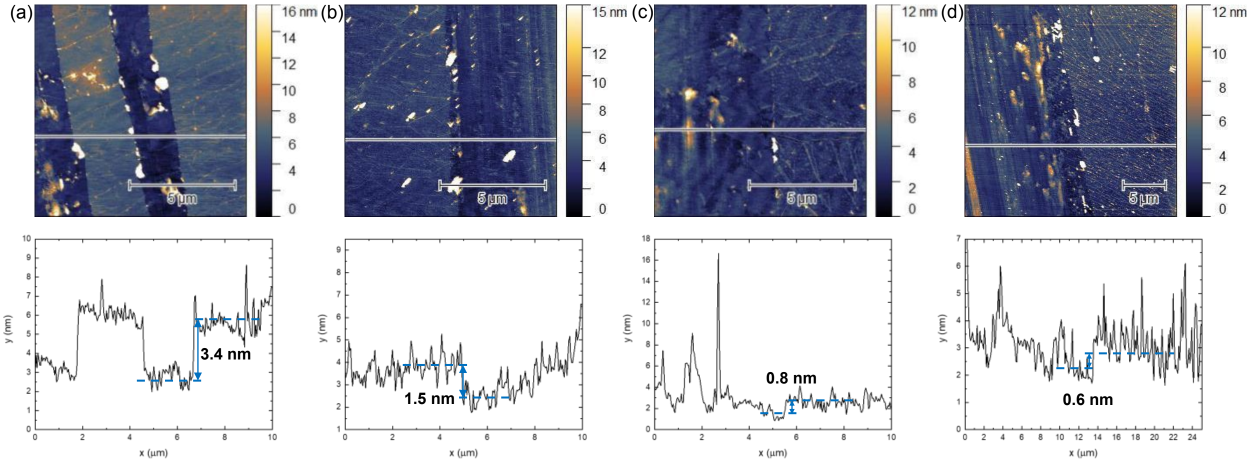

Figure S11. AFM height images (top) and step height profiles (bottom) of PFO-CP polymer films of varying thicknesses: (a) 3.4 nm, (b) 1.5 nm, (c) 0.8 and (d) 0.6 nm (nominal thickness values derived from ellipsometry are 3.6, 1.5, 0.8 and 0.6 nm, respectively) on graphene supported on a Si/SiO_2 substrate. Graphene was exposed by scratching the polymer films with a clean 18G needle.

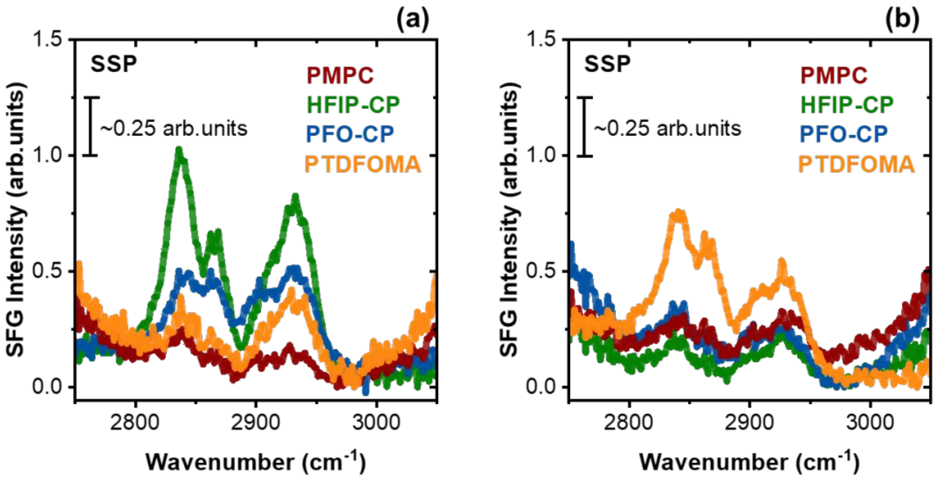

Figure S12. SFG spectra obtained from the polymers deposited on SiO_2 coverslips with a thickness of: (a) 1 µm and (b) ~5 nm.

Fitting Results: ω and Γ are in cm^{-1}, A and χ_{NR} have arbitrary units, and ϕ is in degrees (Resonances 1-C-CH_2 SS, 2-O-CH_2 SS/ N-CH_2 SS, 2-CH_3 SS, 3-CH_3 FR).

Table S2. Fitting parameters for the SFG data obtained with the SSP polarization combination.

**Parameters**	**PMPC**	**HFIP-CP**	**PFO-CP**	**PTDFOMA**
A_1	3.6 ± 3.0			
ω_1	2849 ± 5			
Γ_1	25 ± 13			
A_2	11.9 ± 1.7	11.9 ± 0.9	4.2 ± 1.2	3.1 ± 1.0
ω_2	2900 ± 2	2904 ± 1	2905 ± 4	2903 ± 4
Γ_2	20 ± 2	14 ± 1	15 ± 5	12 ± 4
A_3	4.9 ± 1.6	5.3 ± 1.5		
ω_3	2944 ± 2	2946 ± 2		
Γ_3	15 ± 4	16 ± 4		
χ_{NR}	0.392	0.272	-0.239	0.407
ϕ	141 ± 10	108 ± 6	127 ± 17	135 ± 20

Note: For PTDFOMA, two additional bands were fitted at 2760 cm^{-1} and 3000 cm^{-1} to obtain a better fit curve. A_{2760} = 5.5 ± 4.9, Γ_{2760} = 26 ± 18 and A_{3000} = -6.7 ± 5.4, Γ_{2750} = 35 ± 23.

Fitting Results: ω and Γ are in cm^{-1}, A and χ_{NR} have arbitrary units, and ϕ is in degrees (Resonances 1-C-CH_2 AS, 2-O-CH_2 AS/ N-CH_2 AS, 3-CH_3 AS (in-plane), 4-CH_3 AS (out-of-plane)).

Table S3. Fitting parameters for the SFG data obtained with the PPP polarization combination.

Parameters	PMPC	HFIP-CP	PFO-CP
A_1		1.1 ± 0.9	
ω_1		2895 ± 6	
Γ_1		11 ± 10	
A_2			1.8 ± 1.5
ω_2			2915 ± 7
Γ_2			15 ± 13
A_3	4.0 ± 1.0	3.8 ± 0.6	2.1 ± 1.3
ω_3	2950 ± 4	2950 ± 2	2960 ± 5
Γ_3	12 ± 3	10 ± 2	12 ± 7
A_4	-2.3 ± 1.8		
ω_4	2978 ± 6		
Γ_4	10 ± 7		
χ_{NR}	0.343	0.331	0.349
ϕ	85 ± 26	112 ± 13	166 ± 26

Details on Orientational Analysis

Hyperpolarizability relationships and refractive indices used for orientations analysis of the CH_3 group:[3–6]

$\beta aca \sim \beta ccc = 1$, $\beta aac/\beta ccc = 3.4$

Refractive indices: n_1 (polymer), n_2 (graphene) and n_i (interface)

$n_{1,SFG} = 1.49$, $n_{1,vis} = 1.48$, $n_{1,IR} = 1.48$
$n_{2,SFG} = 2.76$, $n_{2,vis} = 2.40$, $n_{2,IR} = 2.37$
$n_{i,SFG} = 2.12$, $n_{i,vis} = 1.94$, $n_{3,IR} = 1.93$

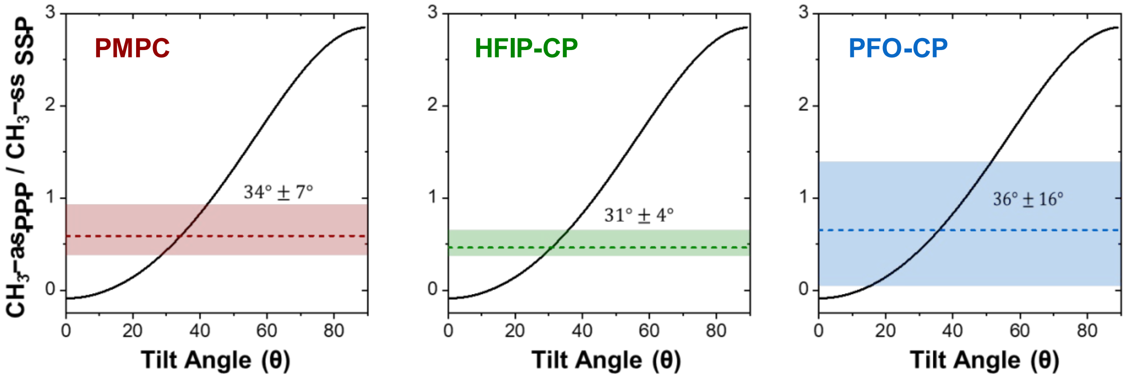

Figure S13. Simulated and experimental (dashed and highlighted region) CH_3–*as*,$_{PPP}$/CH_3–ss,$_{SSP}$ amplitude ratios.

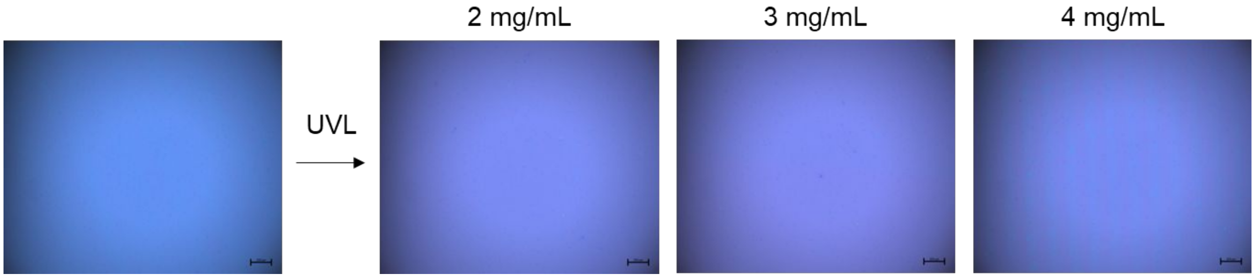

Figure S14. Optical micrographs of HFIP-copolymer films on graphene/SiO_2/Si substrate prepared from varying solution concentrations (2 to 4 mg/mL) before and after UV lithography through a photomask.

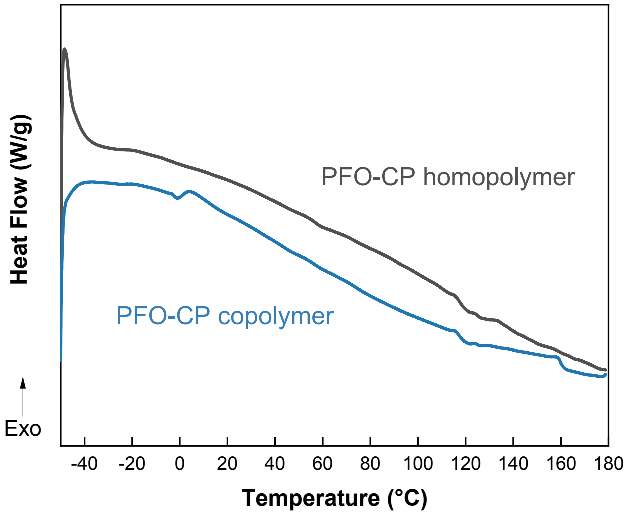

Figure S15. DSC curves of PFO-CP homo- and copolymers obtained from the second heating cycle with a ramp rate of 10 °C/min. The glass transition temperature is determined to be ~117°C for both polymers.

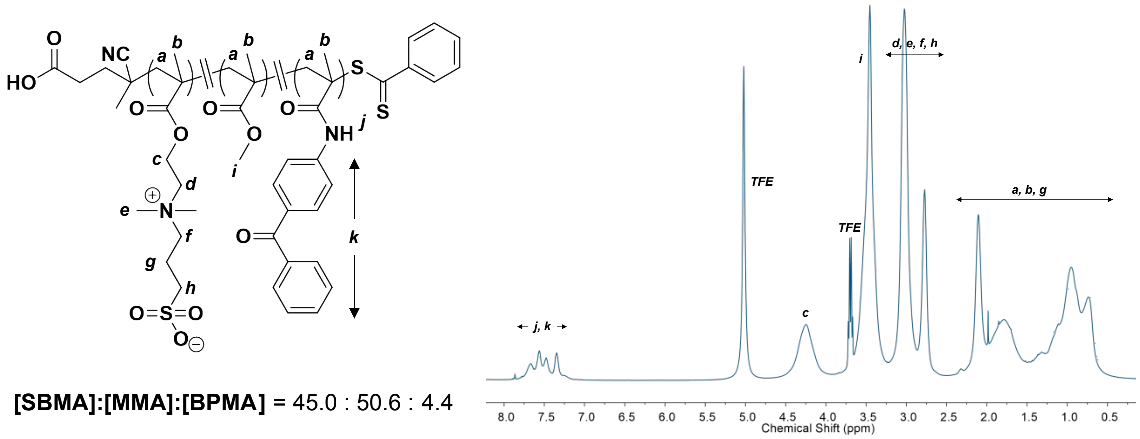

Figure S16. Chemical structure and ^{1}H NMR spectrum of SBMA copolymer with MMA and BPMA (TFE-d$_3$, 500 MHz).

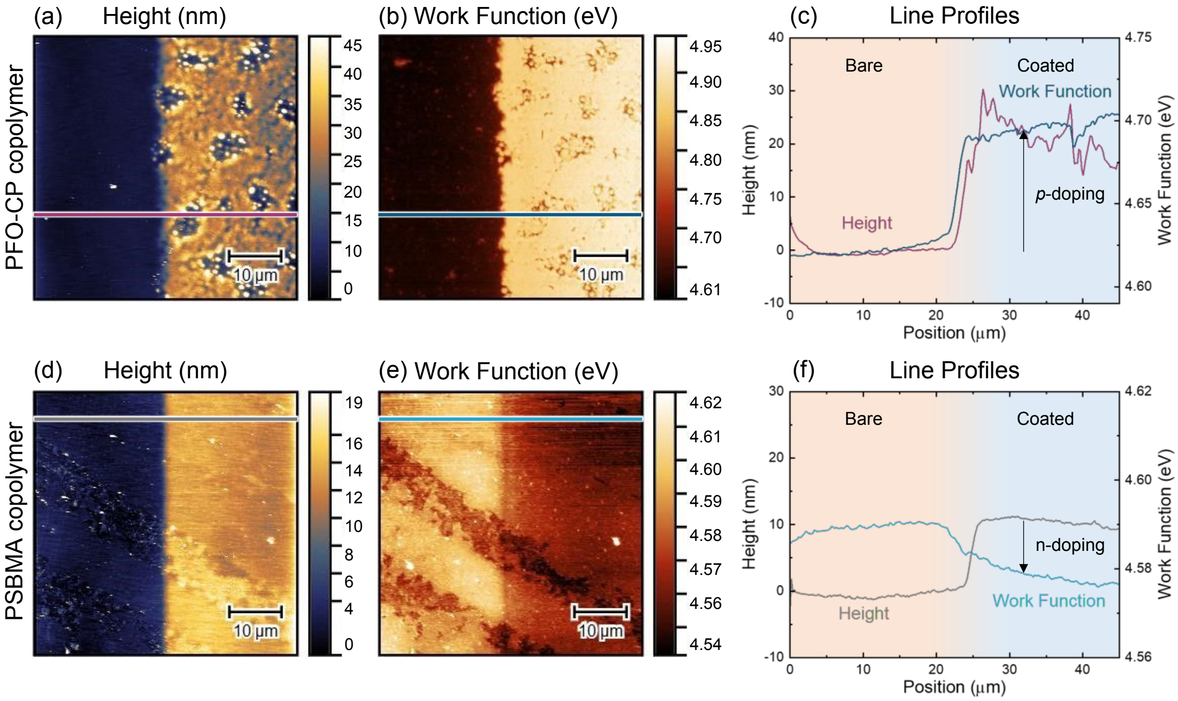

Figure S17. Co-patterned PFO-CP (fluorozwitterist) and PSBMA (zwitterist) copolymers: KPFM of the interface between patterned stripes and bare graphene showing (a,d) height and (b,e) WF with corresponding (c,f) line profiles. The patterned fluorozwitterist- and zwitterist-induced *p*-doping and *n*-doping, respectively, relative to neighboring bare graphene surface.

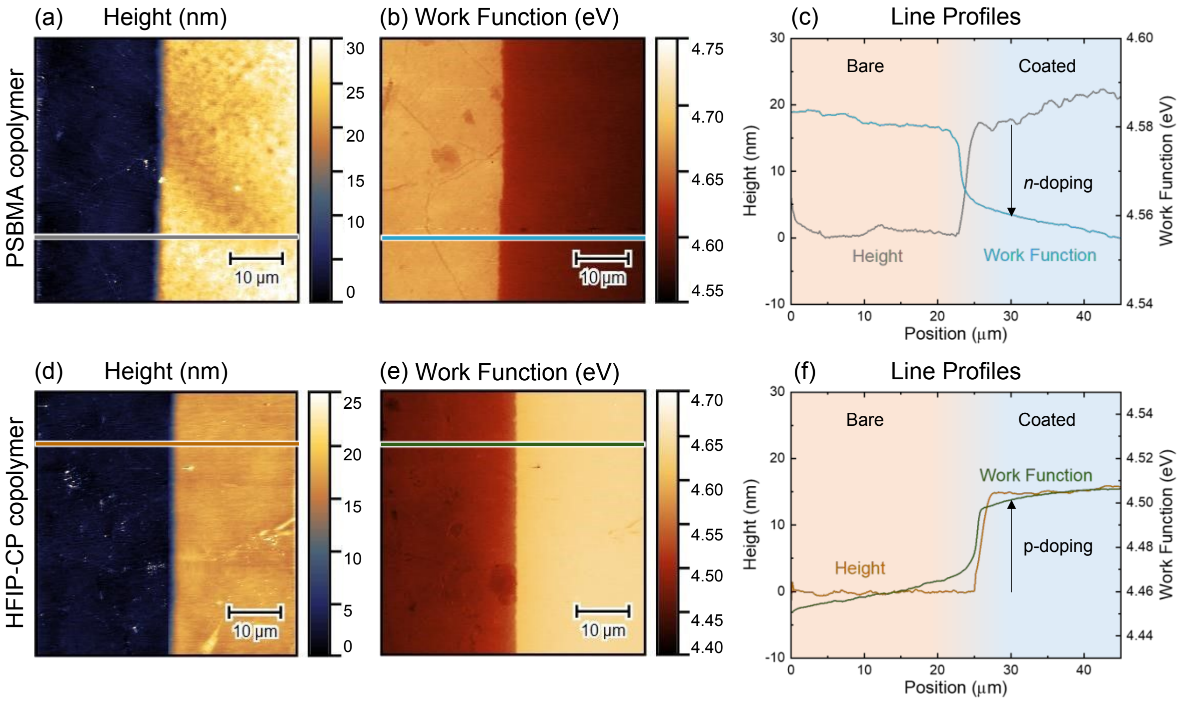

Figure S18. Co-patterned PSBMA (zwitterist) and HFIP-CP (fluorozwitterist) copolymers: KPFM of the interface between patterned stripes and bare graphene showing the (a,d) height and (b,e) work function images with corresponding (c,f) line profiles. The patterned fluorozwitterist and zwitterist induced *p*-doping and *n*-doping, respectively, relative to neighboring bare graphene surface.

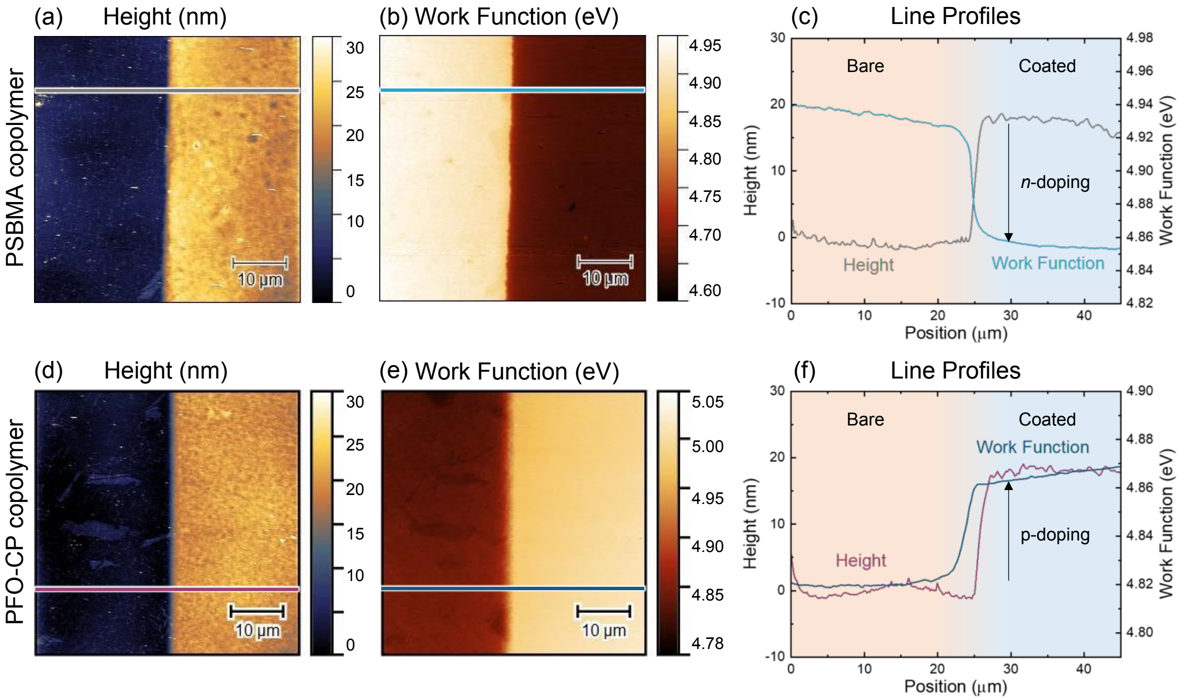

Figure S19. Co-patterned PSBMA (zwitterist) and PFO-CP (fluorozwitterist) copolymers: KPFM of the interface between patterned stripes and bare graphene showing the (a,d) height and (b,e) work function images with corresponding (c,f) line profiles. The patterned fluorozwitterist and zwitterist induced *p*-doping and *n*-doping, respectively, relative to neighboring bare graphene surface.

Table S4. Thickness and work function values of co-patterned polymers.

Patterning Order	$Thickness_{FCP}$ (nm)	$\Delta\phi_{FCP}$ (meV)	$Thickness_{PSBMA}$ (nm)	$\Delta\phi_{PSBMA}$ (meV)
HFIP-CP/PSBMA	12	75	15	−70
PFO-CP/PSBMA	17	80	10	−15
PSBMA/HFIP-CP	22	−25	15	60
PSBMA/PFO-CP	18	−90	20	50

References

(1) Zhou, L.; Triozzi, A.; Figueiredo, M.; Emrick, T. Fluorinated Polymer Zwitterions: Choline Phosphates and Phosphorylcholines. *ACS Macro Lett.* **2021**, *10* (10), 1204–1209.

(2) Yang, Z.; Snyder, D.; Pagaduan, J. N.; Waldman, A.; Crosby, A. J.; Emrick, T. Mesoscale Polymer Surfactants: Photolithographic Production and Localization at Droplet Interfaces. *J. Am. Chem. Soc.* **2022**, *144* (48), 22059–22066.

(3) Tateishi, Y.; Kai, N.; Noguchi, H.; Uosaki, K.; Nagamura, T.; Tanaka, K. Local Conformation of Poly(Methyl Methacrylate) at Nitrogen and Water Interfaces. *Polym. Chem.* **2010**, *1* (3), 303–311.

(4) Chowdhury, A. U.; Chang, D.; Xu, Y.; Hong, K.; Sumpter, B. G.; Carrillo, J.-M. Y.; Doughty, B. Mapping the Interfacial Chemistry and Structure of Partially Fluorinated Bottlebrush Polymers and Their Linear Analogues. *Langmuir* **2021**, *37* (1), 211–218.

(5) Wang, H.-F.; Gan, W.; Lu, R.; Rao, Y.; Wu, B.-H. Quantitative Spectral and Orientational Analysis in Surface Sum Frequency Generation Vibrational Spectroscopy (SFG-VS). *International Reviews in Physical Chemistry* **2005**, *24* (2), 191–256.

(6) Wang, H.-F.; Velarde, L.; Gan, W.; Fu, L. Quantitative Sum-Frequency Generation Vibrational Spectroscopy of Molecular Surfaces and Interfaces: Lineshape, Polarization, and Orientation. *Annual Review of Physical Chemistry* **2015**, *66* (1), 189–216.
